# Differential Involvement of Three Brain Regions during Mouse Skill Learning

**DOI:** 10.1523/ENEURO.0143-19.2019

**Published:** 2019-08-14

**Authors:** Aldis P. Weible, Michael I. Posner, Christopher M. Niell

**Affiliations:** 1 Institute of Neuroscience, University of Oregon Eugene, Oregon, 97403; 2 Department of Psychology, University of Oregon Eugene, Oregon, 97403; 3 Department of Biology, University of Oregon, Eugene, Oregon, 97403

**Keywords:** anterior cingulate cortex, hippocampus, skill learning, visual cortex

## Abstract

Human skill learning is marked by a gradual decrease in reaction time (RT) and errors as the skill is acquired. To better understand the influence of brain areas thought to be involved in skill learning, we trained mice to associate visual-spatial cues with specific motor behaviors for a water reward. Task acquisition occurred over weeks and performance approximated a power function as often found with human skill learning. Using optogenetics we suppressed the primary visual cortex (V1), anterior cingulate cortex (ACC), or dorsal hippocampus (dHC) on 20% of trials at different stages of learning. Intermittent suppression of the V1 greatly reduced task performance on suppressed trials across multiple stages but did not change the overall rate of learning. In accord with some recent models of skill learning, ACC suppression produced higher error rates on suppressed trials throughout learning the skill, with effects intensifying in the later stages. This would suggest that cognitive influences mediated by the anterior cingulate continue throughout learning. Suppression of the hippocampus only modestly affected performance, with largely similar effects seen across stages. These results indicate different degrees of V1, ACC, and dHC involvement in acquisition and performance of this visual-spatial task and that the structures operate in parallel, and not in series, across learning stages.

## Significance Statement

Mice resemble humans with improvements in accuracy and speed during skill learning. Through optogenetics, we can suppress different regions of the mouse brain at different stages of training to better understand when each region contributes to learning. Here we found that primary visual cortex (V1) suppression reduced accuracy across all training stages. Suppressing anterior cingulate cortex (ACC), a region thought to be important for attention early in training, also reduced accuracy throughout learning. Suppressing the hippocampus, a structure critically involved in associative learning, affected performance more modestly. These findings reveal parallel, rather than serial, involvement of these three structures in a mouse model of skill learning.

## Introduction

The study of human skill learning examines both the processes involved in individual trials as well as the stages through which the person passes from initial acquisition to final performance. For example, Sternberg (1969) proposed that an individual trial involved the processes of encoding input, searching memory, selecting the response and executing the response, a sequence that is supported by recent imaging data (Tenison et al., 2016). On the broader scale, Fitts and Posner (1967) proposed that acquiring expertise in a skill involved a progression through cognitive, associative, and autonomous stages. This influential three-stage model has received significant experimental support, and was most recently validated using the ACT-R model developed by John Anderson and associates (Tenison and Anderson, 2016).

According to these models, stages of skill learning progress in serial order. But how do different brain regions engage during learning to facilitate the transition from the novice to the expert? One possibility is that involvement of different brain areas might, like the three-stage model proposed by Fitts and Posner, occur in a strictly serial order. Alternatively, different structures might operate fully in parallel. For example, multiple nodes of the neural circuit might be engaged at the start of training, with connections strengthening over time with trial repetition (Frey and Morris, 1997, 1998; Redondo and Morris, 2011). A third possibility is that, while different regions might be maximally active at different times, their patterns of activation overlap, thus representing a fusion of the serial and parallel models (Formisano and Goebel, 2003; Tenison et al., 2016). The goal of the present study was to compare serial versus parallel involvement of three brain regions, the primary visual cortex (V1), anterior cingulate cortex (ACC), and dorsal hippocampus (dHC) in a mouse model of skill learning.

To accomplish this goal, we trained mice running on a spherical treadmill to respond to the location of a visual stimulus (above or below center on a computer monitor) with a motor action (running to the left or right) for a water reward. This skill requires several weeks for mice to attain mastery. We examined the improvement of the mice across sessions in accuracy, bias, and reaction time (RT) to derive an overall view of learning. We used optogenetic control of parvalbumin-expressing interneurons (PV-INs) to suppress excitatory activity at different stages of training in V1, ACC, and dHC. Because PV-INs provide broad and potent inhibitory control over pyramidal cells, their activation effectively inhibits global levels of excitatory output (Lien and Scanziani, 2011; Atallah et al., 2012). We used a PV-Cre driver line together with a Cre-dependent channelrhodopsin-2 (ChR2) line (Boyden et al., 2005) to provide cell-type specific depolarization with a high degree of temporal specificity. In the PV-ChR2 cross, light delivered through optic fibers increases PV-IN firing, thus suppressing regional excitatory activity.

We first considered how average improvement in performance compared with human skill learning. Next, we sought to determine how each region of interest was involved at different stages of training. We suppressed activity in each of the three brain areas on 20% of the trials during early, middle and late stages of learning based on specific performance criteria, as well as a final over-trained stage. By suppressing performance on only a small number of trials we sought to compare performance between suppressed and unsuppressed trials within stages while not impairing task acquisition.

V1 was selected for suppression because of the use of visual cues in the task. An effect of V1 suppression would confirm that the task was forebrain dependent, a result which would in turn be crucial to interpreting the presence or absence of suppression effects in either of the other two regions. The ACC was selected because it plays a central role in high level attention and top-down cognitive control (Botvinick et al., 2001; Petersen and Posner, 2012), and has been implicated previously in different forms of skill learning and performance (Raichle, 1998; Procyk et al., 2000; Poldrack and Gabrieli, 2001; Karim et al., 2017). The dHC, which also exhibits changes in activation with skill learning (Poldrack and Gabrieli, 2001; Karim et al., 2017), was selected because it is crucial for many forms of associative learning (Brasted et al., 2003; Suzuki, 2007; Squire and Wixted, 2011). Our approach should shed light on whether, when, and how each region is involved in a visual-spatial discrimination mouse model of skill learning.

## Materials and Methods

### Mice

All procedures were conducted in accordance with the ethical guidelines of the National Institutes of Health and were approved by the Institutional Animal Care and Use Committee at the University of Oregon. Animals were maintained on a reverse 12/12 h light/dark cycle. Training and experiments were performed during the dark phase of the cycle. All mice were male, 8–12 weeks of age at the time of surgery, and were bred to the C57Bl6/J background strain. Optogenetic suppression of excitatory activity was performed in offspring (total *n* = 41; V1 *n* = 8, ACC *n* = 9, dHC *n* = 10, control *n* = 14) from a cross between homozygotic Pvalb-IRES-Cre (“PV”, 008069; The Jackson Laboratory) and Rosa26-CAG-LSL-ChR2H134R-eYFP (“ChR2”, Ai32, 012569; The Jackson Laboratory) lines. In these mice (PV-ChR2), ChR2 was expressed in PV-INs.

### Surgery

We administered atropine (0.03 mg/kg) preoperatively to reduce inflammation and respiratory irregularities. Surgical anesthesia was induced and maintained with isoflurane (1.25–2.0%). Optogenetic suppression of neural activity was achieved by passing light through 200 µm in diameter optic fibers implanted bilaterally over the caudal ACC (AP: 0.6 mm, ML: 0.4 mm, DV: 0.4 mm, 15° angle toward the midline), V1 (AP: –3.5 mm, ML: 2.2 mm), or dHC (AP: –2.0 mm, ML: 2.0 mm, DV: 0.8 mm). A titanium cross-bar was also fixed to the skull to enable restraint of the mouse during behavior training. Fibers and cross-bar were cemented in place with Grip Cement. Carpofen (10 mg/kg) was administered postoperatively to minimize discomfort. Mice were housed individually after the surgery and allowed 7 d of postoperative recovery.

### Optogenetic suppression

**Figure 1.**
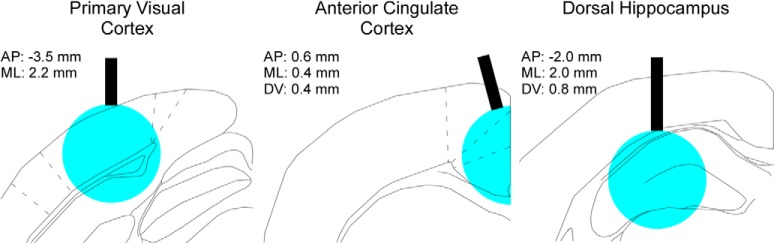
Chronically-implanted optic fibers enable light delivery to V1, ACC, and dHC. Optic fibers (200 µm in diameter; illustrated by the black rectangle) were implanted for delivery of 450-nm wavelength “blue” light to stimulate PV-INs, resulting in the suppression of excitatory output. The expected volume of suppression is a region of tissue 1.5 mm in diameter (illustrated by the blue circle; Weible et al., 2014). AP: anterior (+) or posterior (–) to bregma; DV: ventral to the cortical surface; ML: lateral to the midline.

We suppressed excitatory activity in PV-ChR2 mice using a 450-nm wavelength diode laser set to an output power of 6.3 mW. The resulting intensity of 200 mW/mm^2^, as measured at the tip of the 200 µm in diameter fiber, results in suppression through a volume ∼1.5 mm across (Weible et al., 2014). The expected spread of light through each of the three target regions is illustrated in Figure 1. Rise/fall times of laser pulses were 5 µs. We measured laser power and rise/fall times with a Thorlabs PM100D power meter. Suppression was induced during 20% of trials in a pseudorandomly determined manner. Suppression was initiated at the moment the mouse successfully requested a trial, and continued until a movement was executed (or 10 s had elapsed).

### Behavioral apparatus and training

Mice were trained to perform a visual-spatial discrimination task for a water reward. Training took place on a spherical treadmill based on designs by Dombeck et al. (2007) and Niell and Stryker (2010). Briefly, a hollow polystyrene ball (250 mm in diameter) was set in a 3D printed hemi-spherical base. Air delivered through the bottom of the base was adjusted to a rate of flow enabling free rotation of the ball. A two-tined fork, extending from the back and over the top of the ball, provided the anchor points to fix the mouse in position on the ball. Visual stimuli were presented on a ViewSonic VA2342 liquid crystal display monitor [28 × 50 cm; linearized by eye to correct for gamma (mean luminance 35 cd/m^2^)], set 25 cm in front of the mouse. Water rewards were delivered through a spout positioned immediately beneath the mouth of the mouse. An optical mouse set ∼15° below the horizontal in front of the ball was used to detect movement.

Before training, mice were handled for 3 d, and weights for each mouse were collected to establish a baseline for the water restriction. Weights were subsequently collected daily throughout training, and mice falling below 75% of baseline weight were given supplemental water until weights rose back above the minimum 75% of baseline threshold. Having established baseline weights, mice were then placed on the ball for 2 d of familiarization to the apparatus (day 1: ∼5 min; day 2: ∼30 min).

Task performance required first “requesting” a trial by remaining immobile for 1 s, then responding based on the stimulus by moving the ball to the left or right to receive the water reward. Training was performed in two phases. During the first phase, mice learned to request trials (i.e., remaining immobile for 1 s) by receiving “stop” water rewards. This phase lasted 3 d, with stop rewards decreasing in volume across days. During the second phase, water rewards were given only for correct responses, and were reduced in volume as performance improved to maximize trial count per session. Visual cues determined the correct movement selection. Cues in the present task were 15.5 cm in diameter circles containing vertical or horizontal bars positioned at the top or bottom of the screen. Only the spatial location of the circle was predictive of water reward (bar orientation was irrelevant, and alternated pseudorandomly between horizontal and vertical). Rotation of the ball to the left was rewarded when stimuli were presented at the top of the screen (and to the right for stimuli at the bottom). Errors were signaled by a full-field bright flash (two-fold increase in luminance over gray background) that persisted for 1 s.

### Stages of training

Training progressed through four stages. Stage 1 began with the cessation of stop rewards, when mice were rewarded only for correct responses. Stages 2 and 3 began after mice achieved specific criterion levels of performance. Stage 2 began the day after mice first performed >65% correct responses during a block of 50 consecutive trials. Stage 3 began the day after mice performed >85% correct responses in a 50-trial block, marking the point in training at which mice were considered to have “learned” the task. Following stage 3, the percentage of correction trials was gradually reduced to 0, with no laser suppression. When correction trials reached 0%, suppression trial data were collected for stage 4.

**Figure 2.**
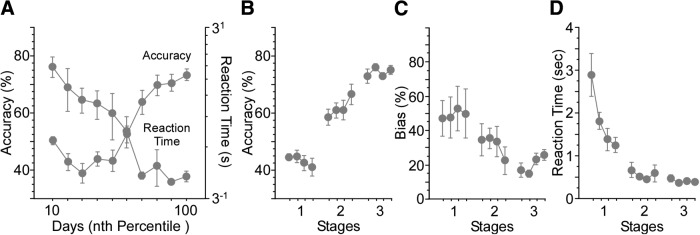
Accuracy and RT performance measures recapitulate changes observed with human skill learning. Control mice acquired the visual spatial discrimination task without interleaved suppression trials. ***A***, The decrease in RT seen as accuracy increases approximates the power function found with human skill learning. ***B–D***, Accuracy, bias, and RT measures are plotted by the 4 d of each stage. Response bias trended lower over the three stages, and RT decreased significantly (bias: df = 11, χ^2^ = 18.5, *p* = 0.07; RT: df = 11, χ^2^ = 61.3, *p* < 0.0001). Analyses for each stage included 4 d of training. Stage 1 included the first 4 d of training. Stage 2 included the first 4 d following performance of 65% correct responses in a 50-trial block. Stage 3 included the 4 d following performance of 85% correct responses in a 50-trial block.

While these stages are somewhat arbitrary, they fit rather closely to mean RT and error data as shown in Figure 2. The mice differed greatly in how many days were required to reach criterion. To plot the average performance we used Vincent curves (Hilgard and Campbell, 1937) which plot the RT and accuracy in each tenth of the overall trials to learning criterion. The first few values in the Vincent curve show marked improvement in RT but accuracy remains at or below chance. This is closely related to what is defined above as the first stage of training. In this stage the association is still unlearned but the general task shows an improvement in RT as different strategies appropriate to the task are selected (e.g., adopting a strong bias, increasing trial count, correctly associating stimulus position with rewarded movement). Later, both RT and accuracy improve. This includes the 65% accuracy defining stage 2 where the mice appear to be learning the correct association while still performing slower than the final RT. Stage 3 is defined by 85% accuracy and corresponds closely to points which show no further improvement in RT or in accuracy, suggesting that performance has reach a kind of asymptote. As defined, stage 4 occurs after mice have reach criterion and correction trials are suspended.

Optogenetic suppression of principal neuron activity was conducted only for the first 4 d of each stage, as this manipulation might impact behavior not only on a trial-by-trial basis, but the overall rate of learning as well. Therefore, each “stage” includes data from 4 d of training. However, achieving each criterion level of performance typically took >4 d of training, and the total days trained varied between mice. Infrequently, mice achieved the next performance criterion in fewer than 4 d. In these cases, mice were considered to have transitioned to the next stage only after the four consecutive days of the previous stage had been completed. In a few instances, mice also failed to learn the task. Mice failing to transition to stage 3 within 50 d were excluded from analysis.

Correction trials, wherein the stimulus on the previous trial was repeated, were used to counter the development of response bias during training. At the start of training, 50% of incorrect responses were followed by a correction trial. This level was maintained or adjusted higher over the course of training, based on the strength of response bias observed, but was never adjusted during the four consecutive days of suppression, as suppression itself could directly bias response selection. To assess the effects of suppression in the absence of correction trials, following the four consecutive days of stage 3, correction trials were gradually reduced to 0%, and mice transitioned to stage 4: four consecutive days of training with interleaved suppression of principal neuron activity in the absence of correction trials. Mice in stage 4 are referred to as over-trained.

To test for effects of suppression on overall rates of learning, data were also collected from control mice that were implanted with fibers but did not undergo suppression during stages 1–3. In this way, we could compare trials-to-learning with and without interleaved suppression. Following the final day of stage 3, however, control mice transitioned to stage 4 with suppression trials. This enabled a comparison to determine whether suppression at different intervals during training impacted effects seen following the removal of correction trials.

### Data analysis

Accuracy data were based on percentage correct responses for suppressed and non-suppressed trials for each session. Analysis of RT (defined as time between visual cue display and mouse movement) data were performed using median RT for each session. To determine bias, we calculated the percentage correct to left (L) and right (R), subtracted each from the percentage correct for the entire session (Session%), and added the absolute value of both.

$${\text{Bias}}_{\text{L},\text{R}}=\text{ Session}\%\;-\;\left(\#{\text{Correct}}_{\text{L},\text{R}}/{\text{ Total}}_{\text{L},\text{R}}\right)$$

$$\text{Total Bias }=\;\left|{\text{Bias}}_{\text{L}}\right|\;+\;\left|{\text{Bias}}_{\text{R}}\right|.$$

The result yields a value where 0% represents no bias and 100% a complete bias to one side.

We chose to use non-parametric analyses in the present study because many of the comparisons involved data that were not normally distributed (verified with the Lilliefors test), and because statistical power is comparable even when the underlying assumptions for the corresponding parametric analysis were met (Kitchen, 2009). Friedman’s test was used to identify within-group effects of suppression across days, as well as changes across days separately for suppressed and non-suppressed trials. *Post hoc* analysis of differences on individual days was performed using a Wilcoxon paired sign test. Between-group comparisons were performed using the Kruskal–Wallis test. To determine whether differences were present between any pair of groups within a given stage (as a *post hoc* analysis of Kruskal–Wallis test results), days within the stage were compared using the Mann–Whitney *U* test.

### Histology

Following stage 4, mice with fibers targeting the ACC or dHC were administered a lethal dose of sodium pentobarbital (100 mg/kg Euthasol) and transcardially perfused with phosphate buffered saline (0.9%) and the 4% paraformaldehyde (PFA). Brains were removed and post-fixed for a minimum of two additional days in 4% PFA, and then sectioned at 100 µm on a vibratome. Fiber placement was then verified with fluorescent microscopy. Two mice were excluded because fibers targeting the hippocampus were found to have terminated either within or beneath the level of the dentate gyrus. All other implanted mice had fiber tracks histologically verified as accurately targeting the ACC or dHC. No attempt was made to histologically verify the placement of fibers targeting V1 as these fibers were placed on the surface on the dura.

## Results

In the present study, we used an animal model of skill learning, a top-bottom visual-spatial discrimination task performed by mice for a water reward, to examine whether and when V1, the ACC, and dHC were involved in task acquisition and performance. Specifically, we examined how optogenetic suppression of these structures influenced movement selection, response bias, and RT.

### Relationship to human skill learning

Before considering the results of our suppression experiments, it is worth addressing whether the mouse data resembles that from human studies. In human skill learning, changes in performance and RT averaged over trials and subjects are often described as fitting a power function (Fitts and Posner, 1967; Newell and Rosenbloom, 1981; Anderson et al., 1999). For RT at least, this feature is recapitulated in mice performing the top-bottom discrimination task. We trained a group of fiber-implanted mice up through stage 3, without suppressing activity in any of the target regions. Figure 2*A* illustrates performance and RT data. Performance data on this scale exhibits a complex pattern, skewed early in training by transient response biases (as illustrated also in Fig. 2*B*,*C*). However, the changes in RT are quite similar to those reported in the human skill learning literature, suggesting at least a superficial similarity over the course of learning.

For the purposes of the present study, we organized the control data into the performance-based training stages used in the suppression experiments below. All of the control mice trained achieved the >85% criterion marking stage 3 (Fig. 2*B*). Response bias trended lower over the three stages, and RT decreased significantly (Friedman’s, bias: df = 11, χ^2^ = 18.5, *p* = 0.07; RT: df = 11, χ^2^ = 61.3, *p* < 0.0001; Fig. 2*C*,*D*).

### Suppression effects by group

**Figure 3.**
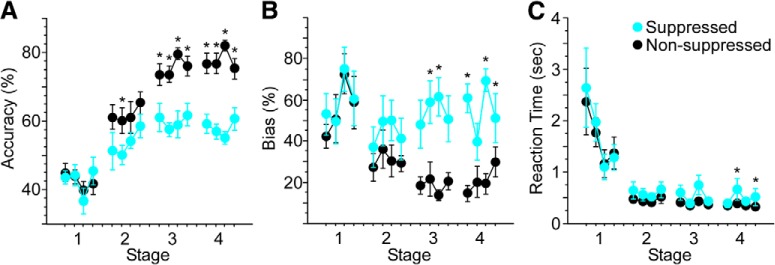
V1 suppression significantly reduces accuracy and increases response bias and RT. Light was delivered to V1 to suppress excitatory activity on 20% of trials through stage 4. ***A***, Suppression reduced accuracy across stages compared with non-suppressed trials (Friedman’s, df = 1, χ^2^ = 30.3, *p* < 0.0001). ***B***, Response bias increased on suppressed versus non-suppressed trials (Friedman’s, df = 1, χ^2^ = 36.1, *p* < 0.0001). ***C***, Suppression produced a small but significant increase in RT across stages (Friedman’s, df = 1, χ^2^ = 6.6, *p* = 0.01). Analyses for each stage included 4 d of training. Stage 1 included the first 4 d of training. Stage 2 included the first 4 d following performance of 65% correct responses in a 50-trial block. Stage 3 included the first 4 d following performance of 85% correct responses in a 50-trial block. Following stage 3, correction trials were reduced gradually to 0%, at which point mice received an additional 4 d of overtraining (stage 4); *p < 0.05, **p < 0.01.

All eight mice undergoing V1 suppression during training successfully acquired the task. Performance on suppressed trials was significantly lower than non-suppressed trials (Friedman’s, df = 1, χ^2^ = 30.3, *p* < 0.0001; Fig. 3*A*). The impact of suppression was initially evident during stage 2, and grew more robust during stages 3 and 4. Suppression also influenced response bias. As illustrated in Figure 3*B*, strong response biases often developed early in training. These were addressed following stage 1 by increasing the percentage of correction trials. As mice learned the relationship between cue position and rewarded direction of movement, biases decreased on non-suppressed trials, exhibiting a strong trend toward significance across all four stages of training (Friedman’s, df = 15, χ^2^ = 25.0, *p* = 0.05). As biases decreased, the percentage of correction trials was reduced. With suppression, however, biases remained elevated (Friedman’s, df = 1, χ^2^ = 36.1, *p* < 0.0001), and were on par with those seen during stage 1. Finally, suppression increased RT. Median RTs decreased significantly across stages for both non-suppressed (Friedman’s, df = 15, χ^2^ = 40.6, *p* = 0.0001) and suppressed (Friedman’s, df = 15, χ^2^ = 29.5, *p* = 0.01) trials. This decrease in RT was modestly, but significantly, less with suppression (Friedman’s, df = 1, χ^2^ = 6.6, *p* = 0.01).

**Figure 4.**
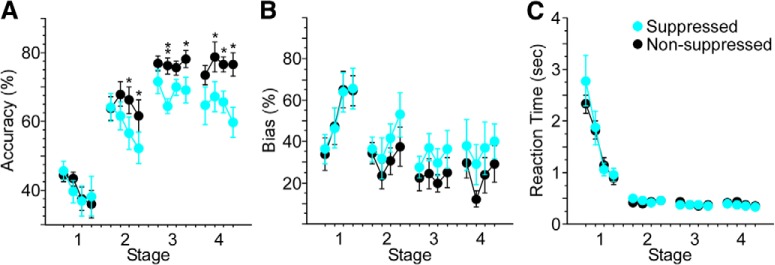
ACC suppression significantly reduces accuracy and increases response bias. Light was delivered to ACC to suppress excitatory activity on 20% of trials through stage 4. ***A***, Suppression reduced accuracy across stages compared with non-suppressed trials (Friedman’s, df = 1, χ^2^ = 9.6, *p* = 0.002). ***B***, Response bias increased on suppressed versus non-suppressed trials (Friedman’s, df = 1, χ^2^ = 4.6, *p* = 0.033). ***C***, Suppression did not significantly affect RT across days of training. Analyses for each stage included 4 d of training. Stage 1 included the first 4 d of training. Stage 2 included the first 4 d following performance of 65% correct responses in a 50-trial block. Stage 3 included the first 4 d following performance of 85% correct responses in a 50-trial block. Following stage 3, correction trials were reduced gradually to 0%, at which point mice received an additional 4 d of overtraining (stage 4); *p < 0.05, **p < 0.01.

All nine mice undergoing ACC suppression during training successfully acquired the task. Performance on suppressed trials was significantly lower than non-suppressed trials (Friedman’s, df = 1, χ^2^ = 9.6, *p* = 0.002; Fig. 4*A*). As with suppression in V1, the impact of ACC suppression was evident first during stage 2, and grew more robust during stages 3 and 4. ACC suppression also influenced response bias (Fig. 4*B*). While biases decreased across days for both non-suppressed and suppressed trials (Friedman’s, df = 15, χ^2^ = 52.3, *p* < 0.0001 and χ^2^ = 32.2, *p* = 0.006, respectively), response bias remained greater with suppression (Friedman’s, df = 1, χ^2^ = 4.6, *p* = 0.033). RT decreased significantly across stages during both non-suppressed and suppressed trials (Friedman’s, df = 15, χ^2^ = 97.2, *p* < 0.0001 and χ^2^ = 96.8, *p* < 0.0001, respectively; Fig. 4*C*), and was not affected by suppression (*p* = 0.54).

**Figure 5.**
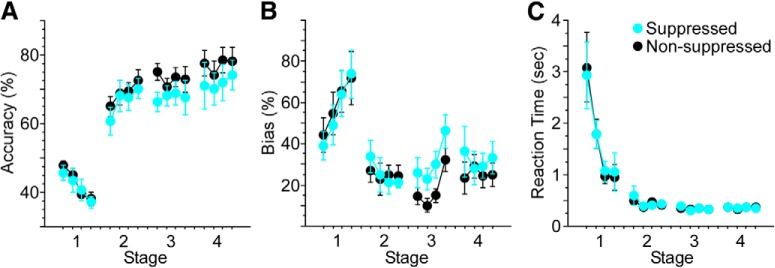
Suppression of dHC modestly reduces response accuracy. Light was delivered to dHC to suppress excitatory activity on 20% of trials through stage 4. ***A***, Suppression reduced accuracy across stages compared with non-suppressed trials (Friedman’s, df = 1, χ^2^ = 4.8, *p* = 0.03). Neither response bias (***B***) nor RT (***C***) changed significantly with suppression. Analyses for each stage included 4 d of training. Stage 1 included the first 4 d of training. Stage 2 included the first 4 d following performance of 65% correct responses in a 50-trial block. Stage 3 included the first 4 d following performance of 85% correct responses in a 50-trial block. Following stage 3, correction trials were reduced gradually to 0%, at which point mice received an additional 4 d of overtraining (stage 4).

Eight of 10 mice undergoing suppression of dHC during training successfully acquired the task (the 2 mice failing to reach stage 3 criterion performance within 50 d of training were excluded from analysis). Performance on suppressed trials was lower than non-suppressed trials across stages (Friedman’s, df = 1, χ^2^ = 4.8, *p* = 0.03; Fig. 5*A*). No differences were observed for individual days (Wilcoxon signed rank). Biases for both non-suppressed and suppressed trials decreased significantly across days (Friedman’s, df = 15, χ^2^ = 41.8, *p* = 0.0002 and χ^2^ = 25.5 and *p* = 0.04, respectively; Fig. 5*B*), with a weak trend toward a difference between conditions (*p* = 0.15). RT for both non-suppressed and suppressed trials decreased significantly across days (Friedman’s, df = 15, χ^2^ = 73.4, *p* < 0.0001 and χ^2^ = 76.9, *p* < 0.0001, respectively; Fig. 5*C*) with no significant difference between conditions (*p* = 0.98).

### Comparisons between groups

**Figure 6.**
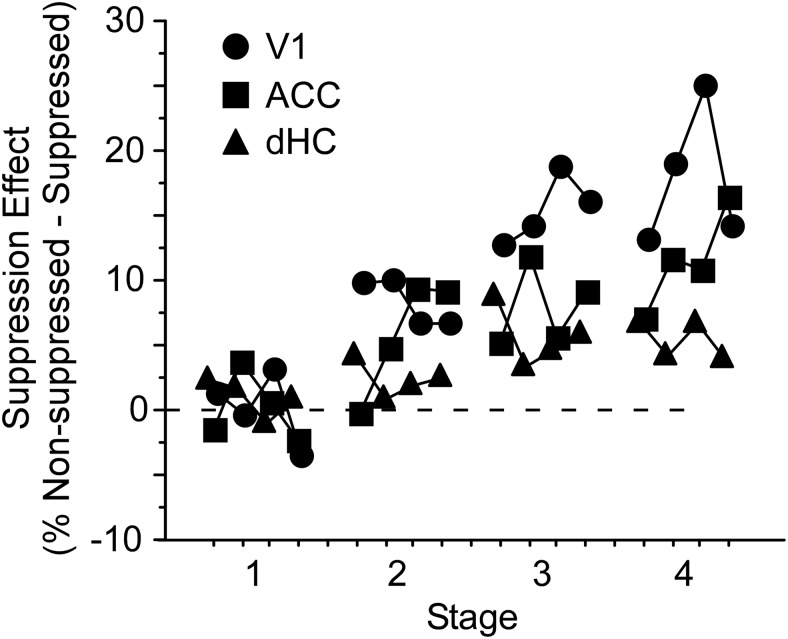
Decreases in accuracy grow over time with suppression of ACC and V1, but not dHC. Suppression effect size was calculated by subtracting accuracy of suppressed trials from non-suppressed trials. The size of the suppression effect increased significantly across days for V1 and ACC mice (Friedman’s, df = 15, χ^2^ = 62.4, *p* < 0.0001 and df = 15, χ^2^ = 25.7, *p* = 0.04, respectively), but not dHC mice. Differences in effect size were significant between groups during stages 3 and 4 (Kruskal–Wallis, df = 2, *p* = 0.002 and df = 2, *p* < 0.0001, respectively), with effect size in V1 exceeding that of ACC during stages 3 and 4 (Mann–Whitney, *z* = –2.7, *p* = 0.006 and *z* = –2.4, *p* = 0.02, respectively), and ACC exceeding that of dHC during stage 4 (Mann–Whitney, *z* = –2.4, *p* = 0.02).

As illustrated in Figures 3–5, the size of the suppression effect on accuracy (accuracy on non-suppressed minus suppressed trials) varied both across days and between groups. Suppression of V1 and ACC had an increasingly large effect on accuracy over time (Friedman’s, df = 15, χ^2^ = 62.4, *p* < 0.0001 and df = 15, χ^2^ = 25.7, *p* = 0.04, respectively; Fig. 6). The effect size with dHC suppression did not change significantly across days (*p* = 0.90).

The difference in effect size between groups that was first apparent during stage 2 grew to significance during stages 3 and 4 (Kruskal–Wallis, df = 2, *p* = 0.002 and df = 2, *p* < 0.0001, respectively). Effects of V1 suppression on accuracy were greater during stages 3 and 4 than those seen with ACC suppression (Mann–Whitney, *z* = –2.7, *p* = 0.006 and *z* = –2.4, *p* = 0.02, respectively), and the size of effect seen with ACC suppression during stage 4 exceeded that seen in dHC (Mann–Whitney, *z* = –2.4, *p* = 0.02). A difference in percentage correction was observed between suppressed groups during stage 3 (Kruskal–Wallis, df = 2, *p* = 0.0002; Table 1). However, this is unlikely to explain the differences in effect size as no correction was employed during stage 4.

**Table 1.** % Correction trials

	V1	ACC	dHC	Control
Stage 1	50	50	50	50
Stage 2	69.5 ± 0.09	65.4 ± 0.02	65.5 ± 0.01	78.1 ± 0.02
Stage 3	47.5 ± 0.01	26.7 ± 0.04	35.8 ± 0.03	30.0 ± 0.03
Stage 4	0	0	0	0

All values % ± SE.

**Figure 7.**
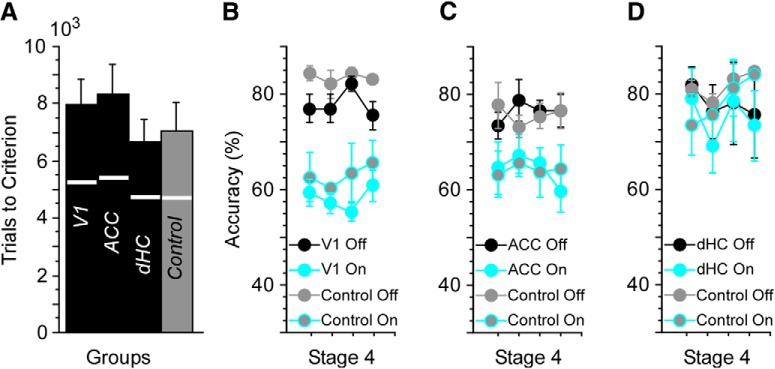
Suppression effects on accuracy are trial specific. ***A***, Control mice and mice undergoing V1, ACC, and dHC suppression learned to stages 2 and 3 criteria in a comparable number of trials (accuracy criteria: >65% and >85% correct responses in a 50-trial block, respectively). Trials to stage 2 indicated by the white line bisecting each bar. Control mice did not undergo suppression over the course of training, but were implanted with fibers targeting V1, ACC, or dHC. During stage 4, suppression was performed in V1, ACC, or dHC mice and compared with effects seen in controls with matching fiber placements undergoing suppression for the first time. The results demonstrate that suppression over the course of training in V1 (***B***), ACC (***C***), or dHC (***D***) did not have a subsequent effect on suppression performed after mice reached the >85% criterion.

While suppression of spiking activity imposed in each of these regions was intended to be trial specific, it is nonetheless possible that this manipulation could have had broader consequences on learning and performance. To test whether suppression impacted rates of acquisition, we compared trials-to-criterion for control mice and V1, ACC, and dHC suppressed mice. No significant effect of group was observed (Kruskal–Wallis, *p* = 0.29; Fig. 7*A*). Accuracy on non-suppressed trials across the first three stages also did not differ between the four groups (Kruskal–Wallis, *p* = 0.83).

These data indicate that trial-specific suppression did not influence rates of acquisition more broadly. It is also possible that the efficacy of suppression might change over time. Because control mice were implanted with fibers, we were able to suppress activity in these mice for the first time at stage 4 and compare the effects with those seen at stage 4 in V1, ACC, and dHC mice (i.e., mice that had undergone suppression during the previous three stages). Suppression of V1 or ACC in control mice significantly reduced accuracy at stage 4 (Friedman’s, df = 1, χ^2^ = 9.1, *p* = 0.004 and df = 1, χ^2^ = 16.9, *p* < 0.0001, respectively). The size of these effects was comparable to those seen with V1 and ACC mice that had undergone suppression through stage 3 (Fig. 7*B*,*C*). Finally, accuracy of control mice was unaffected by suppression of dHC during stage 4 (Fig. 7*D*)

### RT and suppression

In contrast to accuracy and response bias, RT across days only changed with suppression of V1 (Fig. 3). However, RT on any given trial is often influenced by the outcome of the preceding trial (Laming, 1979; Rabbitt, 1966). We therefore examined first the relationship between error trials and RT in the present data, and then tested for any impact of suppression in V1, ACC, and/or dHC following an incorrect choice.

**Figure 8.**
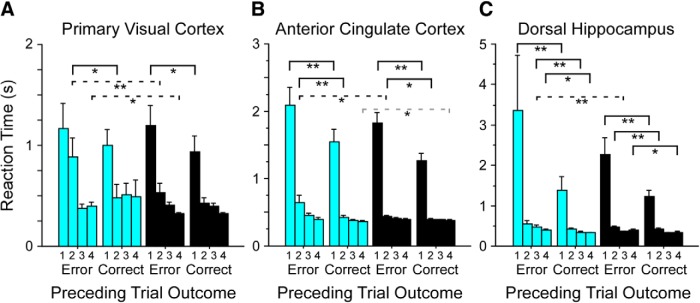
Suppression increases RT following errors. Errors during training were signaled by an increase in screen luminance that persisted for 1 s. RT on correct responses was longer following errors than following correct responses. Suppression further lengthened RTs following errors but had no effect on RT following correct responses. These patterns were observed for mice from each suppression group, with performance on suppressed trials indicated by blue bars, and non-suppressed trials by black bars. ***A***, In V1, suppression lengthened RTs following errors during stages 2 and 4 (dashed black lines; Wilcoxon, *z* = –2.7, *p* = 0.006 and *z* = –2.4, *p* = 0.01, respectively). ***B***, In ACC, suppression produced longer RTs during stage 2 (dashed black line; Wilcoxon, *z* = –2.3, *p* = 0.02). ***C***, In dHC, suppression following an error produced longer RTs during stage 3 (dashed black line; Wilcoxon, *z* = –2.8, *p* = 0.004). Dashed gray line: increased RT on non-suppressed trials. Solid black line: increased RT following error versus correct trials, within either suppressed or non-suppressed conditions; *p < 0.05, *p < 0.01.

We compared correct trial RTs on trial *N* + 1 following correct and incorrect (error) responses on trial *N*. RTs were consistently greater on trial *N* + 1 following an error, for both non-suppressed and suppressed trials (Wilcoxon, *z* = –16.7, *p* < 0.0001 and *z* = –12, *p* < 0.0001, respectively). Suppression lengthened trial *N* + 1 RTs following errors (Wilcoxon, *z* = –4.1, *p* < 0.0001) but had no effect following correct responses (*z* = –0.7, *p* = 0.51). These patterns could be broken down further by region of suppression and stage of training. In V1, suppression lengthened trial *N* + 1 RTs following errors during stage 2 and stage 4 (Wilcoxon, *z* = –2.7, *p* = 0.006 and *z* = –2.4, *p* = 0.01, respectively; Fig. 8*A*, dashed black lines). In ACC, post-error suppression produced longer RTs during stage 2 (Wilcoxon, *z* = –2.3, *p* = 0.02; Fig. 8*B*, dashed black line). In dHC, suppression following an error produced longer RTs during stage 3 (Wilcoxon, *z* = –2.8, *p* = 0.004; Fig. 8*C*, dashed black line). In no region did suppression increase RTs following correct choices. We also examined the effect of suppression during trial N to determine whether mice had a smaller RT during trial *N* + 1 following an error when the error might have been due to suppression. This could happen particularly with suppression of V1 since mice would be less influenced by visual input. However, in no condition did we find that suppression of trial N influenced RT on trial *N* + 1. This may be because the cues introduced after the subject response gave the mice sufficient information about whether they were correct even with suppression.

### Summary

In the present study, we sought to examine serial versus parallel involvement of three brain regions of interest in skill learning, using a visual-spatial discrimination task performed by mice. The impact of suppression of excitatory spiking activity in each region was analyzed during three training stages distinguished by varying levels of accuracy, and a fourth, over-trained stage added here to test the effects of suppression in the absence of correction trials. Mice showed improvements in speed and accuracy over days that were qualitatively similar to changes seen with human skill learning. With suppression, no changes were seen during stage 1. However, effects seen during stages 2 and 3 resembled a reversion to stage 1, suggesting that the absence of a stage 1 effect reflects a behavioral floor in performance. Suppression of V1 reduced accuracy and enhanced bias, effects that increased across stages. This increase is likely attributable to the overall improvement in performance, with the result that mice had further to drop back toward the behavioral floor. Suppression of ACC produced effects on accuracy and bias that were qualitatively similar to those seen in V1, but of lesser magnitude. Effects of suppression on accuracy in dHC mice were modest in comparison to those seen in V1 and ACC mice, and appeared to be largely restricted to stage 3. An overall effect on RT was only observed with suppression in V1. This effect was modest, and most evident in the late stage of training (Fig. 3). However, RTs were consistently longer following errors, and this effect was enhanced with suppression in all three regions during different stages of training.

## Discussion

The goal of the present study was to compare serial versus parallel involvement of three brain regions, the V1, ACC, and dHC, in a mouse model of skill learning. Suppression of excitatory activity in V1 and ACC significantly decreased accuracy and increased response bias across stages of training. Similar, though less robust, effects were observed with dHC suppression. While overall effects on RT were only seen with V1 suppression, RTs were lengthened with suppression in all three regions following errors at different stages of training. Taken together, these data are generally consistent with a model of parallel involvement of these three regions in our visual-spatial discrimination mouse model of skill learning.

### Cortical areas

We examined the effects of V1 suppression as a control to determine whether cortical processing was necessary for the visual-spatial discrimination. We assumed that suppression of the visual system would influence performance throughout the task. The ACC and dHC, both of which have been found to exhibit changes in activation with skill learning and performance (Raichle, 1998; Procyk et al., 2000; Poldrack and Gabrieli, 2001; Karim et al., 2017), were also hypothesized to play a role based on specific task features. Performance of the visual-spatial discrimination required mice to transform vertically-oriented (top-bottom) cues to horizontal (left-right) movements. An action based on this type of transformation is more cognitively demanding than one involving a cue directing movement in the same plane and direction (e.g., a leftward cue directing a leftward movement). In addition to transforming cue orientation to plane of movement, mice also had to inhibit any pre-existing natural bias which could conflict with the appropriate movement. Because the ACC is involved in higher-level attention (Petersen and Posner, 2012) and conflict resolution (Botvinick et al., 2001) we hypothesized that it would be involved in the early stage of the task as mice sought the optimal strategy for reward. Our task also required forming numerous different associations. Because the dHC is critically involved in associative learning (Brasted et al., 2003; Suzuki, 2007; Squire and Wixted, 2011), we hypothesized that it would be involved during early and middle stages as the associations among stimulus location, movement, and reward delivery were developed and strengthened. While our task is conceptually simple, mice clearly found forming and acting on the associations specific to the optimal strategy challenging as evidenced by the thousands of trials required to reach criterion performance. This lends further credence to a proposed role for the dHC in our task as forming even the simplest associations may require the hippocampus if stimulus parameters are changed to increase task difficulty (Beylin et al., 2001).

### Serial and parallel views

Both serial and parallel models of performance of individual trials during skill learning have been proposed. Sternberg (1969) provided a serial model and developed the additive factors method for determining which independent variables influenced any of the successive stages needed to carry out the task. Anderson and colleagues (Anderson, 2005; Tenison et al., 2016) have developed serial models using ACT-R to simulate complex problem solving tasks. Parallel models of individual trials often use a race horse analogy in which responses compete and the first to reach a threshold determines the response (Logan, 2002; Ratcliff et al., 2016). Because stored items accumulated over time, later trials were faster than earlier ones. Logan’s model, however, argued for a continuous process, not for stages that overlapped in time.

Most cognitive models of the stages of learning have used a serial viewpoint to describe the stages involved. Fitts and Posner proposed that skill learning progressed through three stages (Fitts and Posner, 1967; Tenison et al., 2016). The first stage was cognitive and involved evaluation of conflicting strategies in accord with past experience. The second stage was associative and involved connecting the strategies into an overall solution. The third stage was autonomous and involved practice to achieve a highly efficient program to execute the skill. The likely reason that such a serial process has been widely used is that early and late parts of the learning curve clearly do not overlap.

Data from the present study are consistent with a parallel view of information processing across brain areas during skill learning. Nevertheless, behavioral changes in the present study were reminiscent of those one might expect based on the serial three-stage model proposed by Fitts and Posner. For example, different strategies are available to mice to obtain water rewards, and the effectiveness of some of these strategies depends on external factors including reward size and the percentage of correction trials. Before stage 1, mice are rewarded simply for standing still on the ball. At the start of stage 1, this shaping step is removed, and mice must first request a trial (stand still) and then execute a movement. Only when the movement is in the correct direction will a reward be delivered. At this point, mice typically engage a bias strategy, as seen during stage 1 in Figures 3–5. This phenomenon is both consistent and robust, with 76% of mice from the present study developing a >50% bias during stage 1, and 64% exhibiting biases between 75% and 100%. The experimenter gradually increases the % correction, such that the bias strategy become increasingly inefficient for obtaining water rewards. It is, however, still possible to obtain sufficient water simply by executing more trials. In extreme cases, highly biased mice may engage this second strategy and execute 1000+ trials in a single session. We combat this by decreasing the reward size. At some point, the strategy shifts to one of correctly executing movements based on cue position. Bias, trial count, and % correction trials all are decreased as accuracy increases past 65% in a 50-trial block. Therefore, stage 1 in the present study includes a period during which there are several conflicting strategies available for obtaining the water reward, and the selection of the optimal strategy typically only follows rejection of less efficient ones based on previous experience. Stage 2 includes the period when we first see the specific associations of the optimal strategy executed effectively to achieve the reward. Finally, stage 3 includes the highly efficient and reliable performance of the optimal strategy.

The cognitive stage 1 of Fitts and Posner is generally considered a period when task goals are recognized and that information is used to develop an appropriate sequence of actions for achieving those goals, a process seen as involving conscious evaluation of explicit knowledge. Whether a mouse can be said to engage in such a process cannot be directly ascertained from the current data. However, this question is secondary to our demonstration that suppression of V1, ACC, and dHC impact behavior throughout training in this mouse model of skill learning.

Evidence in the present study of involvement of different regions across different stages of learning is not unexpected. Using multivoxel neuroimaging techniques Tenison et al. (2016) showed it was possible to examine the extent to which several brain areas were active during stages of a problem-solving task. While prefrontal areas were most active during the early stage, and motor areas in the late stage, there was overlap between the neural areas and the stages. This is consistent with other neuroimaging (Formisano and Goebel, 2003) and animal studies (Redondo and Morris, 2011) that have shown that brain areas overlap with respect to their involvement in the learning process, supporting a parallel view of information processing. But how might we interpret the effects observed in the present study, especially those associated with suppression of the ACC and dHC?

Effects of ACC suppression mirrored the time course of V1 suppression, decreasing accuracy throughout training. While our data are consistent with a parallel model, it is possible that the ACC performs different functions at different stages of training which may, or may not, directly relate to skill learning. The ACC is clearly involved in attention and top-down control, processes that would be critical early in training in the identification and adoption of an optimal strategy for task performance (Botvinick et al., 2001; Petersen and Posner, 2012). At the other end of the spectrum, the ACC has been implicated in recall of remote memories (Bontempi et al., 1999), with the time course for learning in the present study easily accommodating that required for the structural changes seen in the ACC accompanying memory consolidation (Vetere et al., 2011). Potentially representing a bridge between these two roles, Procyk et al. (2000) described trial-to-trial transitions in the primate ACC between search-related activity involving increased attentional load and repetition-related activity based on memory of a motor program when attention to action was reduced. While their study does not speak to skill learning per se (all motor behaviors and task solutions had been learned before recording), it demonstrates responses of different neurons in the same region associated with either attention or recall. It is possible that the route and/or direction of information flow also change with stage of learning. The ACC connects with the hippocampus through both the thalamus (Xu and Sudhof, 2013) and entorhinal cortex (Anderson et al., 2016). Information also travels in both directions between the ACC and dHC. Determining whether and how these observations relate to ACC involvement throughout training, could be addressed by future studies.

While dHC suppression did modestly reduce accuracy, it did not affect the overall rate of acquisition. The relatively small effect of hippocampal suppression may support the idea that learning in this task involves the striatum rather than the hippocampus (Makino et al., 2016). Suppression may also have been more effective were it to have been performed on a higher percentage of trials. It is also possible that the root of the modest effects seen here are methodological in nature. Based on the literature, PV-IN stimulation should be an effective means to suppress principal neuron activity in the hippocampus. Hippocampal PV-INs have extensive axonal arbors yielding an enormous number of synaptic contacts (Sik et al., 1995; Norenberg et al., 2010). Stimulation of PV-INs expressing ChR2 effectively suppresses neighboring CA1 pyramidal neuron activity (Ognjanovski et al., 2017). Thus, we are confident that this approach accomplished the desired physiologic effect in the hippocampus within the illuminated region. We cannot, however, fully discount the complex geometry of the dHC, and the possibility that targeting multiple sites along the septal-temporal axis may have yielded more robust effects. Regardless, our dHC data are in line with a parallel model where the computations in each brain area occurred throughout learning rather than being specific to any stage.

A major unanticipated result of this study was that, while suppression decreased accuracy, it had relatively little overall effect on RT. This was a surprising result since increasing task difficulty and distracting attention have been shown to increase RT and error in both rodents and humans (Beane and Marrocco, 2004; for review, see Posner and Niell, 2019). Manipulating neural activity through optogenetics is, of course, not something done in humans, thus complicating the comparison. However, less invasive efforts to interfere with network performance by transcranial magnetic stimulation (Capotosto et al., 2009) or electrical stimulation (Zmigrod et al., 2016) show increases in RT during performance in humans.

It should not be concluded that RT is an insensitive measure in the mice. As shown in Figure 2, RT declines regularly with practice in mice much as in humans. Moreover, mice show longer RTs following error than following a correct response. This effect was even greater when the error occurred during a suppressed trial in a stage-dependent and region-dependent manner (Fig. 8*B*,*D*). In humans, slowing following an error even when there is no explicit error feedback is often taken as evidence that the person was aware of the error (Wessel, 2012). However, our experimental design differed from many human studies in a critical way that may explain the effects we observed: errors triggered a distinct visual cue (a full-field bright flash), which may have made the error salient to the mice. However, as we do not have physiologic data necessary to determine whether the error trials were accompanied by the error related negativity found in humans, directly testing this hypothesis is beyond the scope of the present study (Gehring et al., 1995, 2018).

In the present study, the goal was to “suppress” activity in different regions of the brain to examine how each was involved in skill learning and performance at different stages of proficiency. The direct effect of the laser was confined to the specific area (e.g., V1). However, an important caveat in the interpretation of optogenetic effects is that the impact on overall brain activity is not limited to the region of direct inactivation. Unlike other lesion methods, the optogenetic suppression is nearly immediate, giving no time for other areas of the brain to adjust to the loss of the suppressed areas. For example, the suppression of activity in V1 is propagated upstream and downstream to other brain areas, resulting in perturbation of these areas that may be greater than just the loss of visual input. Thus, the temporal specificity of action and maintenance of structural integrity seen as major advantages of optogenetics over conventional techniques may in fact expose adjacent elements of the circuit to unintended disruption (Hong et al., 2018). One potential alternative interpretation of the effect seen with ACC suppression on performance involves the direct, reciprocal projections linking the ACC and V1 (Zhang et al., 2014). Effects of ACC and V1 suppression in the present study were qualitatively similar. It is possible that ACC suppression impacted behavior indirectly through the V1 projection, resulting in the similar, albeit smaller, impairment in accuracy and increased bias. It is worth noting, however, that RT did not increase with suppression of the ACC as it did with suppression of V1, suggesting that the observed similarities may be correlative and not causative. Assuming the effects on performance of ACC or V1 suppression were not due to their interconnections, we believe the most likely effect of suppression of V1 is to impair the visual coding of the stimulus. Mice have “requested” a trial, but do not see a cue, and thus resort to guessing, an interpretation that is consistent with the chance level of performance reported. In contrast, the smaller performance decrement seen with ACC suppression suggests that the cue is visible, but that suppression causes a lapse of attention (Petersen and Posner, 2012) or decrease in salience (Seeley et al., 2007; Menon, 2015; Chen et al., 2016; Peters et al., 2016) and thus a reversion to responding in the previously preferred (biased) direction.

Two of 10 mice with fibers targeting the dHC failed to learn within 50 d of training, whereas all mice from ACC, V1, and control groups successfully acquired the task. It seems unlikely that these two mice failed to acquire the task because of the optogenetic suppression, as the effects observed in the remaining eight were the smallest among the three groups undergoing suppression during training, and mean trials to criterion was the lowest for all four groups. It also is unlikely that the failure was attributable to the greater invasiveness of the surgical procedure (fibers targeting the dHC were implanted deeper than those targeting the ACC or V1). In addition to the eight mice that learned from this group, an additional five control mice with dHC fibers successfully acquired the task. We believe, therefore, that the most parsimonious explanation for the failure of these two mice to acquire the task was some reason other than the optogenetic manipulation or the surgery itself, and that it is simply chance that both mice were from one group.

Overall, our evidence supports that our mouse skill learning task is greatly influenced by V1 and ACC throughout learning. It shows a more modest influence from the suppression of the dHC. Notably, inactivating the ACC influences errors without a change in RT. Since this last effect is different from would be expected based on data from humans it requires further studies to explore its significance.

## Synthesis

Reviewing Editor: Morgan Barense, University of Toronto Decisions are customarily a result of the Reviewing Editor and the peer reviewers coming together and discussing their recommendations until a consensus is reached. When revisions are invited, a fact-based synthesis statement explaining their decision and outlining what is needed to prepare a revision will be listed below. The following reviewer(s) agreed to reveal their identity: Taraz Lee.

The ability to suppress specific brain regions at a trial by trial level offers a very exciting tool for studying the involvement of different brain regions in skill acquisition. The study design was well thought out, and considered and tested several interesting alternative hypotheses. While at times the mix of regions and stages were difficult to follow, on a whole, we felt that the authors did a good job of communicating the results of their work.

Our main critiques of this research are with regard to some assumptions that underlie the study. Below we discuss the assumptions we identified, and some challenges we have to those assumptions.

Assumption 1: Stages of learning are correctly identified

One of our main concerns with this paper is the assumption that the stages identified with accuracy cut-offs are accurately identifying the three stages described in the Fitts and Posner theory of skill acquisition. The 3 phases of skill acquisition are distinguished in humans by a shift in the cognitive processes used to perform a task. These shifts are associated with a reduction in the speed it takes to perform a task, due to the specialization of productions and strengthening of associations. The improvements in accuracy that accompany skill acquisition are an even more indirect measure of these processing shifts. For this reason, typically models of skill acquisition within humans focus on reaction time in order to generate a more accurate estimation of what phase people are in. In this paper the authors distinguish the stages with accuracy cut offs: Stage 1 is until mice perform >65% correct in 50 consecutive trials, Stage 2 ends when mice reach >85% at which point they transition to Stage 3. Baring a more complicated stage identification method such as used in Tenison & Anderson (2015) or Anglim and Wynton (2015), the authors need to justify why these cutoffs offer a rough estimate of when mice shift in the type of cognitive processing they are using to complete the task.

As the authors mention, performance is at floor during all of stage 1. It would be impossible to examine any effects of stimulation on accuracy and there really isn't any evidence for skill learning during this stage. The choice of 65% and 85% for stages 2 and 3 seem arbitrary and are not well motivated. A reporting of the mean days to criterion at each stage would also be useful.

Assumption 2: Interference at the trial level will have impacts on both performance and learning.

The authors do discuss this assumption briefly in the discussion, and we think this is an important point to consider. The negative impact of suppression at the trial level suggests that suppression interferes with task execution. The authors use cutoffs to test is there are differences in the suppression effect across the stages they identified to determine if the processing demands change. Rather than rely on accurately setting cut offs the authors could look at whether there are distinct shifts in the impact that suppression has on performance (accuracy, RT, and bias). Such an analysis would need to account for differences in mice and other factors of the task, and may be beyond the scope of the current study.

Additionally, we are interested in the mechanism the authors hypothesize would lead suppression of these regions to impact learning. Are suppressed trials, trials that mice cannot learn from? Do we have evidence in humans that a higher error rate during learning correlates with a shallower learning curve with relation to RT? One interpretation of the N+1 analysis (Line 344) is that errors or suppression events might actually be supporting a different learning mechanism than the associative strengthening that we likely see with correct trials. Stahl and Feigenson (2015) describe the role of unexpected events to trigger infant's learning and exploration. Might the suppression of regions be encouraging more attention to the encoding of the problem on the following trial? While answering this is outside the scope of this study, a more explicit discussion of the learning processes the authors hypothesize are being used by the mice in this task and the impact of suppression either directly or indirectly on this process would help situate this work with theories of skill acquisition and learning.

Assumption 3: This task engages the ‘cognitive phase’ in the first place.

How realistic is it to assume that the mice are consciously evaluating “strategies for solving the problem and building a goal tree or hierarchy of goal states”? The task appears to cue the mouse with a perceptual stimulus to trigger a motor response which is met with feedback (water or bright screen). We wonder whether there is evidence that mice are engaged problem solving for such a seemingly simple task. As we see it the mouse already has the skills to perform the task (encode, turn left, turn right) at the start of the experiment. What they are learning is an association between stimulus and response, which fits more into what we would describe as the second phase of the three phase model. This would reframe this study not as an investigation into the 3 phase model but as an investigation of the transition from retrieval to automatization, a process which is driven by associative learning. Does reconsidering these results through this lens change the conclusions?

Other comments:

We would have appreciated a more thorough review of why these three brain regions were chosen, and evidence from the literature to support the hypotheses the authors had concerning their role in skill acquisition and task performance. As the authors state, the task was a visual discrimination task requiring intact perception. It is unclear why and how the role of V1 would evolve over the course of learning and how any set of results would shed light on theories of skill learning. The ACC was chosen because of its role in “high-level attention”, but there was no up-front discussion of any prior work examining how activity in this region changes over the course of skill acquisition. Similarly, one might have predicted that the hippocampus might be involved early on in learning given its role in declarative memory and that prior work in rodents has shown its importance for the early stages of learning in tasks such as plus-mazes. The autonomous stage is thought to be when the task has been fully proceduralized and we would have expected a test for the role of the dorsolateral striatum in producing habitual behavior. Although we appreciate the authors have limited time and resources a more thorough explanation for the regions chosen and fleshed out hypotheses for the effects of inactivation would have been helpful.

It seems relatively clear that ACC and V1 suppression had similar detrimental effects on performance at all stages of learning. It is unclear what processes the authors think are affected in these two cases. There needs to a more thorough discussion placing these results in the context of prior work.

It seems notable that 20% of mice undergoing suppression of hippocampal activity failed to acquire the task. Given that all other animals in all groups learned the task, does this not suggest a role for the hippocampus in early skill acquisition?

Minor comments:

line 46 - When initially discussing the power function include a discussion of what it is that decreases according to a power function (e.g. speed of task completion, error rate, etc). Also given that the cause for the power function is a puzzle it may be worth discussing some of the main theories explaining this. This will help tie in the stages of skill acquisition with the power law (e.g. the power law is due to changes in both the type of cognitive processes used to perform a task as well as the speed at which those processes are executed).

line 54 - This is the first mention of a parallel model. What theory is this referring to? Up to this point only a sequential model of skill acquisition has been reviewed. If the intention is to refer to Logan's Race Model it would be worth discussing this. However, I'm not sure if this test would provide a means for testing the difference between these theories.

Line 233 - While historically RT data have been averaged over trials in human studies of the power law more recent work has shown that this approach is flawed and can produce a power-law despite the fact individual participant data does not fit a power law. While it is fine to graph the averaged data, using this to draw conclusions about differences in learning rate or when different phases are met is problematic. Given this challenge, we suggest reframing the ‘Relationship to Human Skill Learning’ to focus more on the evidence of learning within the mice, because as it stands this section does not adequately test the relationship to human skill learning.

Line 276-- Reaction time was not affected by suppression - this is surprising, why do they think this did not impact RT?

Line 327 - This is an interesting comparison between the non-suppressed mice and the suppressed mice.

Line 377 - “larger number of errors during the later stage due to suppression may have resulted from the higher level of performance” (we found this phrasing unclear)... it seems like the errors would be linked to the type of cognitive processes being relied on to do the task, if as mice are acquiring the task they are shifting how they perform the task which makes them more or less susceptible to regions being suppressed. Is this the point the authors are trying to make?

Line 407 - validate seems like a strong word to use here. Would introduce or propose be more accurate?

## Author Response

Dear Dr. Barense,

We appreciate the opportunity to submit a revised version of our manuscript “Differential Involvement of Three Brain Regions During Mouse Skill Learning”. We believe we have addressed the insightful comments made by yourself and the reviewers, and that the manuscript is substantially improved for having done so. The comments were helpful in clarifying both our paper and our thoughts. We hope that the changes we have made will answer the comments and clarify our paper.

Below is a point-by-point response (indented) to the original comments (in italics). Changes to the manuscript text are in bold, as requested.

Sincerely,

The Authors

Computational Neuroscience Model Code Accessibility Comments for Author (Required):

N/A

Significance Statement Comments for Author (Required):

Some minor comments:

Line 26: effected should be affected Corrected

Line 26: delete “on” in the following: “...its influence was on strongest in middle and late learning stages.” Corrected

Comments on the Visual Abstract for Author (Required):

N/A

Synthesis Statement for Author (Required):

The ability to suppress specific brain regions at a trial by trial level offers a very exciting tool for studying the involvement of different brain regions in skill acquisition. The study design was well thought out, and considered and tested several interesting alternative hypotheses. While at times the mix of regions and stages were difficult to follow, on a whole, we felt that the authors did a good job of communicating the results of their work.

Assumption 1: Stages of learning are correctly identified

One of our main concerns with this paper is the assumption that the stages identified with accuracy cut-offs are accurately identifying the three stages described in the Fitts and Posner theory of skill acquisition. The 3 phases of skill acquisition are distinguished in humans by a shift in the cognitive processes used to perform a task. These shifts are associated with a reduction in the speed it takes to perform a task, due to the specialization of productions and strengthening of associations. The improvements in accuracy that accompany skill acquisition are an even more indirect measure of these processing shifts. For this reason, typically models of skill acquisition within humans focus on reaction time in order to generate a more accurate estimation of what phase people are in. In this paper the authors distinguish the stages with accuracy cut offs: Stage 1 is until mice perform >65% correct in 50 consecutive trials, Stage 2 ends when mice reach >85% at which point they transition to Stage 3. Baring a more complicated stage identification method such as used in Tenison & Anderson (2015) or Anglim and Wynton (2015), the authors need to justify why these cutoffs offer a rough estimate of when mice shift in the type of cognitive processing they are using to complete the task.

It is true that the serial stages in the average data of the mice were mostly arbitrary in defining what appears in Figure 1 as a power function with both RT and accuracy. However, these stages are roughly equivalent to an analysis of the relation of accuracy and speed to total trials shown in Figure 1. We have outlined this relationship in a new section called Stages of training (lines 158-182). We hope this shows that our arbitrary division are close to appropriate stages based on both accuracy and RT.

We have also added text to the Discussion intended to relate how the literature maps on to our application of stages to mouse learning (lines 423-432) and how the data validate the selection of the performance-based criteria. (Lines 464-479).

As the authors mention, performance is at floor during all of stage 1. It would be impossible to examine any effects of stimulation on accuracy and there really isn't any evidence for skill learning during this stage. The choice of 65% and 85% for stages 2 and 3 seem arbitrary and are not well motivated. A reporting of the mean days to criterion at each stage would also be useful.

We have discussed the nature of our stages above and in lines 158-182. Figure 7A (shown below) has been modified to indicate the trials to criterion (horizontal line across each bar in accord with your request).

Assumption 2: Interference at the trial level will have impacts on both performance and learning.

The authors do discuss this assumption briefly in the discussion, and we think this is an important point to consider. The negative impact of suppression at the trial level suggests that suppression interferes with task execution. The authors use cutoffs to test is there are differences in the suppression effect across the stages they identified to determine if the processing demands change. Rather than rely on accurately setting cut offs the authors could look at whether there are distinct shifts in the impact that suppression has on performance (accuracy, RT, and bias). Such an analysis would need to account for differences in mice and other factors of the task, and may be beyond the scope of the current study

This is a valid alternative approach to addressing the question. Regrettably, it is not feasible to address the question with the data in hand as we only suppressed activity during the first four days of each stage. To effectively test the approach indicated here would have required suppressing activity on 20% of trials across all days of training. It is, therefore, beyond the scope of the current study as pointed out by the reviewers

Additionally, we are interested in the mechanism the authors hypothesize would lead suppression of these regions to impact learning. Are suppressed trials, trials that mice cannot learn from? Do we have evidence in humans that a higher error rate during learning correlates with a shallower learning curve with relation to RT? One interpretation of the N+1 analysis (Line 344) is that errors or suppression events might actually be supporting a different learning mechanism than the associative strengthening that we likely see with correct trials. Stahl and Feigenson (2015) describe the role of unexpected events to trigger infant's learning and exploration. Might the suppression of regions be encouraging more attention to the encoding of the problem on the following trial? While answering this is outside the scope of this study, a more explicit discussion of the learning processes the authors hypothesize are being used by the mice in this task and the impact of suppression either directly or indirectly on this process would help situate this work with theories of skill acquisition and learning.

As we 1) did not find and effect of suppression on the overall rate of learning, 2) did not have a specific mechanism in mind that might cause such an effect, and 3) specifically tailored the experiment to minimize such an effect (suppressing on only 20% of trials), we have de-emphasized this as a hypothesized outcome. (Lines 76-79, 83-87)

Even though the manipulation did not change the rate of learning, we were still able to determine if suppression was influencing performance during each of the stages by comparing accuracy on suppressed and non-suppressed trials.

We have also expanded parts of the manuscript that discuss how our manipulations and the observed effects might relate to what is known about these structures in the literature. (Lines 487-490, 495-503)

Assumption 3: This task engages the ‘cognitive phase’ in the first place.

How realistic is it to assume that the mice are consciously evaluating “strategies for solving the problem and building a goal tree or hierarchy of goal states”? The task appears to cue the mouse with a perceptual stimulus to trigger a motor response which is met with feedback (water or bright screen). We wonder whether there is evidence that mice are engaged problem solving for such a seemingly simple task. As we see it the mouse already has the skills to perform the task (encode, turn left, turn right) at the start of the experiment. What they are learning is an association between stimulus and response, which fits more into what we would describe as the second phase of the three phase model. This would reframe this study not as an investigation into the 3 phase model but as an investigation of the transition from retrieval to automatization, a process which is driven by associative learning. Does reconsidering these results through this lens change the conclusions?

Although some writing in cognitive psychology identifies the ACC with conscious intent this is not a necessary feature. Interference can influence RT even when the item is masked. The interference effect such as occurs with incompatible responses (a left move for a bottom target) really defines what the ACC does in human information processing. This provides a close link between mouse and human attention (Lines 423-426). As noted above, mouse behavior also provides evidence for strategy competition early in training, which we discuss in detail (464-479).

Other comments

We would have appreciated a more thorough review of why these three brain regions were chosen, and evidence from the literature to support the hypotheses the authors had concerning their role in skill acquisition and task performance. As the authors state, the task was a visual discrimination task requiring intact perception. It is unclear why and how the role of V1 would evolve over the course of learning and how any set of results would shed light on theories of skill learning. The ACC was chosen because of its role in “high-level attention”, but there was no up-front discussion of any prior work examining how activity in this region changes over the course of skill acquisition. Similarly, one might have predicted that the hippocampus might be involved early on in learning given its role in declarative memory and that prior work in rodents has shown its importance for the early stages of learning in tasks such as plus-mazes. The autonomous stage is thought to be when the task has been fully proceduralized and we would have expected a test for the role of the dorsolateral striatum in producing habitual behavior. Although we appreciate the authors have limited time and resources a more thorough explanation for the regions chosen and fleshed out hypotheses for the effects of inactivation would have been helpful.

Suppression of V1 was initially conceived of as a control. It was included at each of the stages to demonstrate that visual cortical processing was involved in perceiving the visual stimulus at the different stages of the task. Were it not, that would raise the possibility that acquisition and performance of the task were not cortically mediated. (Lines 53-54, 414-417)

We have now outlined in detail why the three areas of the brain were chosen. We moved this to the start of discussion section because of the length limit of the Introduction and called this section Cortical Areas. (Lines 412-439)

It seems relatively clear that ACC and V1 suppression had similar detrimental effects on performance at all stages of learning. It is unclear what processes the authors think are affected in these two cases. There needs to a more thorough discussion placing these results in the context of prior work

The effects of ACC and V1 suppression are “similar” in the sense that both manipulations impact accuracy and bias at the same stages. However, there are some quantitative differences as well that may reflect differences in the functional roles of the two structures. V1 suppression appears to be causing the mouse to guess, which may in fact be what is occurring as the suppression may be occluding the visual cue. Accuracy is reduced almost to chance, and bias returns to Stage 1 levels: knowing that it has requested a trial, but having no cue upon which to act due to the suppression, the mouse is reverting to its innate bias to drive response selection. This interpretation is consistent with the minimal effect on RT, as the mouse still knows how to respond quickly, and the potential value of doing so. In contrast, ACC suppression effects are smaller in magnitude. These effects may be indicative of a loss of focus, a distraction, produced by acute ACC suppression. However, the fact that accuracy is still well above chance suggests that the cue remains perceptible, in contrast to what appears to result from V1 suppression.

We expanded on our view of the most likely theory of suppression in V1 and ACC in the Discussion. (Lines 496-507, 566-575)

It seems notable that 20% of mice undergoing suppression of hippocampal activity failed to acquire the task. Given that all other animals in all groups learned the task, does this not suggest a role for the hippocampus in early skill acquisition?

This is a valid point worthy of additional comment in the manuscript. We believe the failure of these two mice to acquire the task would require greater consideration were we also to have seen effects of dHC suppression at least on par with those observed in ACC and V1 mice. However, suppression in these mice had in fact little to no impact on accuracy, bias, or reaction time. Another possible explanation is the degree of invasiveness of the dHC implants compared with those in ACC and V1. Fibers targeting dHC are implanted at a depth of 0.8mm, compared with 0.4mm for ACC and 0.0mm for V1. It is possible that the surgical trauma was greater in these mice, due to implant depth, and that this could have contributed. However, not only did the other 8 of 10 mice from this group successfully acquire the task, but the control mice implanted with fibers into dHC all also acquired the task. In an ongoing study comparing the effects of dorsal versus ventral HC suppression, where fibers targeting vHC penetrate to a depth of 2.0-2.5mm, mice are also able to acquire the task. We believe, therefore, the most parsimonious explanation for the failure of these two mice to acquire the task were for reasons other than the optogenetic manipulation or the surgery itself, and that it is simply chance that both mice were from one group. We have added a paragraph to the Discussion addressing this issue. (Lines 577-588)

MINOR COMMENTS

line 46 - When initially discussing the power function include a discussion of what it is that decreases according to a power function (e.g. speed of task completion, error rate, etc). Also given that the cause for the power function is a puzzle it may be worth discussing some of the main theories explaining this. This will help tie in the stages of skill acquisition with the power law (e.g. the power law is due to changes in both the type of cognitive processes used to perform a task as well as the speed at which those processes are executed).

line 54 - This is the first mention of a parallel model. What theory is this referring to? Up to this point only a sequential model of skill acquisition has been reviewed. If the intention is to refer to Logan's Race Model it would be worth discussing this. However, I'm not sure if this test would provide a means for testing the difference between these theories.

The two comments above are closely related. While we now mention serial and parallel models in the Introduction, because of word limitations we do not elaborate until the Discussion section. We have added material to the Introduction and Discussion to address these issues (Lines 45-49, 441-462)

Line 233 - While historically RT data have been averaged over trials in human studies of the power law more recent work has shown that this approach is flawed and can produce a power-law despite the fact individual participant data does not fit a power law. While it is fine to graph the averaged data, using this to draw conclusions about differences in learning rate or when different phases are met is problematic. Given this challenge, we suggest reframing the ‘Relationship to Human Skill Learning’ to focus more on the evidence of learning within the mice, because as it stands this section does not adequately test the relationship to human skill learning.

This point is well-taken and certainly valid. Data from individual humans do sometimes show what may be interpreted as discrete shifts in the learning curve that may in turn reflect transitions in strategy or degree of proficiency. However, the trial-to-trial and day-to-day variability in mouse data make a comparable analysis problematic at best. Thus, we focused on the data at the group level. We have, however, shifted somewhat away from the “relationship to human skill learning”, instead relating what we have learned in mice, and what is known from the literature, to what is seen with human group data and the relevance to human learning where appropriate. The figure below shows even more clearly how animal attention relates in detail to human results.

Line 276-- Reaction time was not affected by suppression - this is surprising, why do they think this did not impact RT?

We agree that this is a surprising outcome of our study. Usually RT is more sensitive than errors. We do not know the cause of this result. (Lines 405-406)

Line 327 - This is an interesting comparison between the non-suppressed mice and the suppressed mice.

We agree, thank you.

Line 377 - “larger number of errors during the later stage due to suppression may have resulted from the higher level of performance” (we found this phrasing unclear)... it seems like the errors would be linked to the type of cognitive processes being relied on to do the task, if as mice are acquiring the task they are shifting how they perform the task which makes them more or less

This has been clarified in line 398-399

Line 407 - validate seems like a strong word to use here. Would introduce or propose be more accurate?

Changed to “support using”. (Line 411)
